# Biochemical Alterations in Triticale Seedlings Pretreated with Selective Herbicide and Subjected to Drought or Waterlogging Stress

**DOI:** 10.3390/plants12152803

**Published:** 2023-07-28

**Authors:** Zornitsa Katerova, Dessislava Todorova, Elena Shopova, Liliana Brankova, Ljudmila Dimitrova, Margarita Petrakova, Iskren Sergiev

**Affiliations:** Institute of Plant Physiology and Genetics, Bulgarian Academy of Sciences, Acad G. Bonchev Str., Bl. 21, 1113 Sofia, Bulgaria; zornitsa@bio21.bas.bg (Z.K.); dessita@bio21.bas.bg (D.T.); kostei@abv.bg (E.S.); lbrankova@abv.bg (L.B.); dim.lyudmila@gmail.com (L.D.); margarita.p02@abv.bg (M.P.)

**Keywords:** antioxidants, Serrate^®^, stress markers, triticale, water stress, xenobiotic-detoxifying enzymes

## Abstract

Waterlogging and drought disrupt crop development and productivity. Triticale is known to be relatively tolerant to different stress factors. In natural conditions, plants are rather subjected to multiple environmental factors. Serrate^®^ (Syngenta) is a systemic selective herbicide suitable for cereal crops such as triticale and wheat to restrain annual grass and broadleaf weeds. Triticale (×*Triticosecale* Wittm., cv. Rozhen) was grown as soil culture under controlled conditions. Seventeen-day-old plantlets were leaf sprayed with Serrate^®^. The water stress (drought or waterlogging) was applied after 72 h for 7 days, and then the seedlings were left for recovery. The herbicide does not provoke sharp alterations in the antioxidant state (stress markers level, and antioxidant and xenobiotic-detoxifying enzymes activity). The water stresses and combined treatments enhanced significantly the content of stress markers (malondialdehyde, proline, hydrogen peroxide), non-enzymatic (total phenolics and thiol groups-containing compounds), and enzymatic (activities of superoxide dismutase, catalase, guaiacol peroxidase, glutathione reductase) antioxidants, and xenobiotic-detoxifying enzymes (activities of glutathione S-transferase, NADPH:cytochrome P450 reductase, NADH:cytochrome *b5* reductase). These effects were more severely expressed after the drought stress, suggesting that this cultivar is more tolerant to waterlogging than to drought stress.

## 1. Introduction

Triticale (×*Triticosecale* Wittm.) is a crop, artificially obtained after the hybridization of wheat (*Triticum* spp.) with rye (*Secale cereale* L.), which became commercially available approximately a half century ago [1,2]. It is assumed to be relatively tolerant to environmental challenges, due to its rye traits, and it has high genetic diversity for abiotic stress responses [2,3,4]. Drought and waterlogging are among the devastating abiotic stresses, which influence negatively crop yield [5]. Water stresses (drought and waterlogging) negatively affect photosynthesis, lead to redox imbalance and oxidative stress, alter optimal physiological and biochemical processes and ultimately reduce crop yield [6,7,8]. These stresses were studied in detail when applied individually in both field and laboratory conditions [9]. In natural conditions, plants are subjected to combinations of different factors, but the information for the effects is insufficient [10,11]. There are few articles reporting the effect of abiotic stress combination (including herbicides) on the biochemical status of crops [12,13,14,15,16,17]. Generally, the resultant effect of multiple treatments is specific and unpredictable [9,18,19]. The information if the effect of treatment combinations on plants is negative (cross-synergism) or positive (cross-adaptation) could be obtained experimentally.

Among other specific physiological alterations, environmental stresses force plants to intensify accumulation of different reactive oxygen species (ROS) [6,20,21]. Depending on their types and levels in plants, at low concentrations ROS may trigger specific biochemical signals and alterations and could function as signaling molecules, while at high concentrations they cause oxidative stress. Plants respond to rising ROS through well-organized antioxidative defense, integrating enzymatic system (superoxide dismutase (SOD), catalase (CAT), peroxidases, glutathione reductase (GR), glutathione S-transferase (GST), etc.) and non-enzymatic scavengers (glutathione, ascorbate, phenolics, etc.) to keep the optimal cellular redox state. Based on their results Raisat et al. [22] suggested that enzymatic antioxidants might be used to screen for drought tolerant triticale genotypes. The NADPH:cytochrome P450 reductase (CPR) and NADH:cytochrome *b5* reductase (B5R) also take part in defensive mechanisms of plants against abiotic stress [23,24,25]. CPR is a membrane-bound flavoprotein, localized mainly in the endoplasmic reticulum and its function is to transfer reducing equivalents from NADPH to a variety of P450 monooxygenases, involved in secondary metabolism reactions (biosynthesis of phenylpropanoids, terpenoids, alkaloids, fatty acids, signaling molecules, plant hormones, etc.) [24,25,26]. B5R is also localized in the endoplasmic reticulum, but transfers electrons from NADH to cytochrome b5 and next on different lipid-modification reactions as fatty-acid and sterol precursors desaturation, fatty acids hydroxylation, and in P450-interposed reactions [27,28]. In addition, CPR and B5R, together with glutathione and GST, are involved in Phase I and Phase II of xenobiotic catabolism, including herbicide detoxification [26].

Spontaneous weed growth is undesirable in farmlands used for crop cultivation. Nowadays, different chemical compounds and their combinations are utilized in arable land to control this process. Serrate^®^ (Syngenta) contains the inhibitor of fatty acid biosynthesis–clodinafop-propargyl (prop-2-ynyl(R)-2-[4-(5-chloro-3-fluoro-2-pyridyloxy) phenoxy]propionate) together with the inhibitor of amino acid biosynthesis–pyroxsulam (([N-(5,7-dimethoxy[1,2,4]triazolo [1,5-a]pyrimidin-2-yl)-2-methoxy-4-(trifluoro methyl)pyridine-3-sulfonamide])), and a herbicide safener, cloquintocet-mexyl ((RS)-1-methylhexyl [(5-chloro-8-quinolyl)oxy]acetate). This herbicide formulation aims to control annual grass and broadleaf weeds due to its efficacy in cereal crop protection [29]. Serrate^®^ is systemic and selective for triticale, wheat and rye, which are tolerant to its application. The reports concerning triticale subjected to multiple abiotic factors containing water stresses does not include herbicides [19,30,31,32,33].

Previously, the combined effect of Serrate^®^ and subsequent water stress (soil drought or waterlogging) was studied in wheat plants, [13,14,15,34]. Serrate^®^ application was documented to provoke a synergistic physiological effect in seedlings subjected to waterlogging, whereas the biochemical response of wheat was only modulated under drought [13,14]. The current study explores the effect of the same treatment combinations in triticale, as it could be expected to be more tolerant to abiotic stressors than wheat. The study is important in the view of the climate change leading to unstable and extreme weather phenomena (including water stress) and the associated global search for resilient cereals [35,36].

This study aimed to assess the effect of pre-treatment with the selective herbicide Serrate^®^ and subsequent water stress (drought or waterlogging) on the physiological response of triticale by the examination of oxidative stress markers and selected enzymatic and non-enzymatic antioxidants.

## 2. Results

### 2.1. Stress Marker Contents: Hydrogen Peroxide, Malondialdehyde and Proline

To evaluate the effects of water stress the following stress marker contents were monitored: hydrogen peroxide, malondialdehyde and proline.

**Figure 1:**
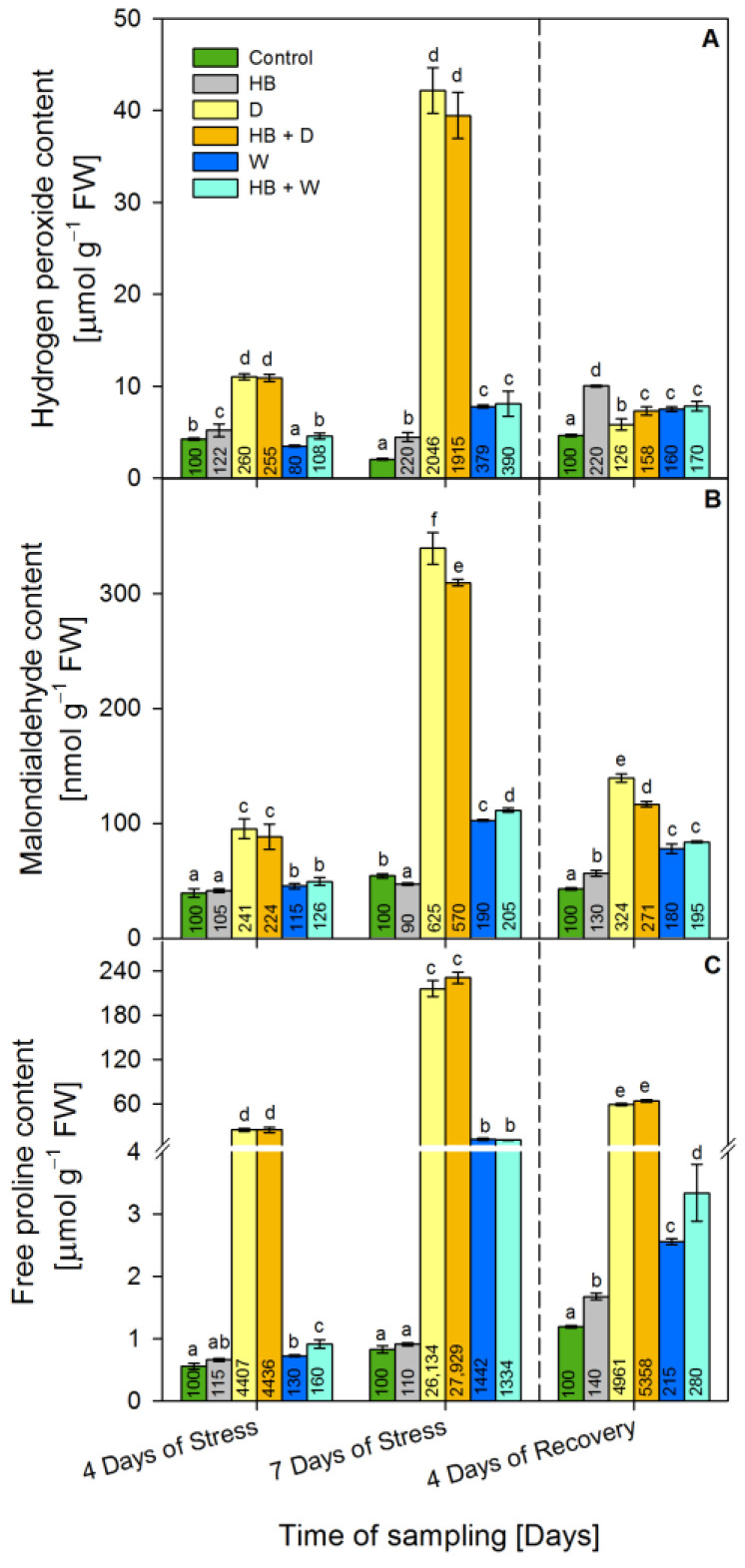
Content of hydrogen peroxide (**A**), malondialdehyde (**B**) and free proline (**C**) in the leaves of triticale treated with selective herbicide (HB) and exposed to waterlogging (W) or drought (D). The numbers in each bar represent % to the respective controls. The statistical significance between treatments at *p* ≤ 0.05 is indicated by different small letters within the column groups.

The hydrogen peroxide (Figure 1A) concentration was raised (by 120%) at the 7th day of stress and at the 4th day of recovery due to the Serrate^®^ application. Drought increased it substantially by 160% (at the 4th day of stress) and by 2046% (at the 7th day of stress) reaching a value of 42.1 µmol g^−1^ FW, but recovery H_2_O_2_ levels reverted to 26% above the respective control. The combined stress (HB + D) caused similar alterations, but at recovery, the H_2_O_2_ level was reverted to 60% above the respective control. Waterlogging slightly decreased (by 20%) the H_2_O_2_ concentrations at the 4th day of stress but then by an increment of 280% at the 7th day of stress and 60% at the recovery was observed. Similarly, the combined stress (HB + W) increased the concentration of H_2_O_2_ to 290% (at the 7th day of stress), and at recovery, the level of H_2_O_2_ reverted to 70% above the respective control.

The herbicide induced moderate alterations in MDA concentrations (Figure 1B): a reduction by 10% (at the 7th day of stress) and an increment of 30% at recovery. Drought applied alone or in combination with herbicide (HB + D) increased considerably MDA levels: by 525% (339.3 nmol g^−1^ FW after drought), by 470% (309.4 nmol g^−1^ FW after H + D) at the 7th day of stress, by 224% (139.5 nmol g^−1^ FW after drought) and by 171% (116.6 nmol g^−1^ FW after H + D) at recovery. Waterlogging had a weaker effect than drought: MDA concentrations rose to 90% at the 7th day of stress and by 80% at recovery. Combined stress (HB + W) altered the MDA levels similarly to waterlogging alone.

Herbicide application raised proline levels by 40% only at the recovery stage (Figure 1C). Drought increased severely proline concentrations as follows: by 4307% (24.8 µmol g^−1^ FW at the 4th day of stress), by 26,034% (215.9 µmol g^−1^ FW at the 7th day of stress) by 4861% (59.0 µmol g^−1^ FW at recovery) in drought-treated seedlings, by 4336% (24.9 µmol g^−1^ FW at the 4th day of stress), by 27,829% (230.8 µmol g^−1^ FW at the 7th day of stress) and by 5258% (63.7 µmol g^−1^ FW at recovery) in triticale subjected to combined (HB + D) treatment. 

Waterlogging also raised the proline levels by 30% (at the 4th day of stress), by 1342% (11.9 µmol g^−1^ FW at the 7th day of stress) but at recovery it reverted to 2.6 µmol g^−1^ FW (115% above the control). Similarly, the combined stress (HB + W) increased proline concentrations by 60% (at the 4th day of stress), by 1234% (11.0 µmol g^−1^ FW at the 7th day of stress) and at recovery to 180% above the respective control.

### 2.2. Non-Enzymatic Antioxidants: Total Phenolics and Thiol Groups Containing Compounds

**Figure 2:**
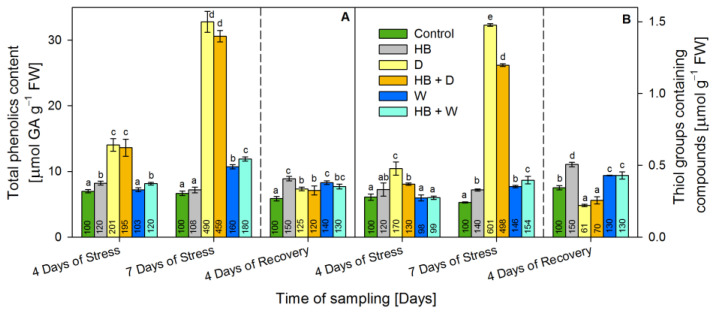
Content of total phenolics (**A**) and thiol groups containing compounds (**B**) in the leaves of triticale treated with selective herbicide (HB) and exposed to waterlogging (W) or drought (D). The numbers in each bar represent % to the respective controls. The statistical significance between treatments at *p* ≤ 0.05 is indicated by different small letters within the column groups.

The herbicide increased the phenolics content (Figure 2A) by 20% at the 4th day of stress and by 50% at recovery. Drought applied alone or in combination with herbicide (HB + D) raised the phenolics gradually to 390% and 360%, respectively, after 7 days of stress, and at recovery, they were reverted to about 20% above the respective control. Phenolics were increased by 60% at the 7th day of stress due to waterlogging, and then were reduced to 40% above the respective control at recovery. These compounds were raised (by 20% and 80%, respectively at the 4th and 7th days) due to the combined treatment (HB + W); then they were reverted to 30% above the respective control at recovery.

Serrate^®^ increased thiols by 40% at the 7th day of stress and by 50% (at the 4th day of recovery above the respective control (Figure 2B). Thiol levels raised during drought treatment by 70% at the 4th day of stress and by 500% at the 7th day of stress, but at recovery, a decline by 40% below the respective control was observed. A similar tendency was found due to the combined treatment (HB + D): an increase by 30% and 400% at the 4th and 7th days of stress, respectively, and a decrease by 30% at recovery. Waterlogging and combined treatment (HB + W) increased thiol groups containing compounds by approximately 50% at the 7th day of stress and by 30% at recovery above the respective control.

### 2.3. Activity of Enzymatic Antioxidants

**Figure 3:**
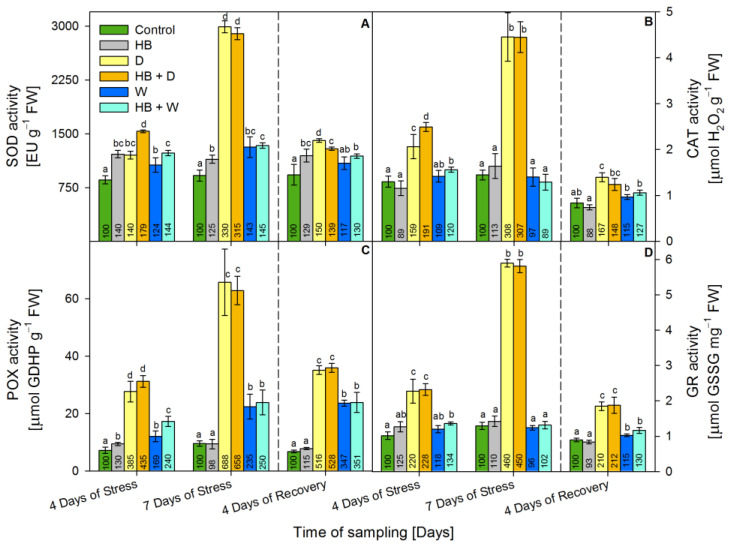
Activity of superoxide dismutase (SOD) (**A**), catalase (CAT) (**B**), guaiacol peroxidase (POX) (**C**), and glutathione reductase (GR) (**D**) in the leaves of triticale treated with selective herbicide (HB) and exposed to waterlogging (W) or drought (D). The numbers in each bar represent % to the respective controls. The statistical significance between treatments at *p* ≤ 0.05 is indicated by different small letters within the column groups.

Serrate^®^ slightly induced SOD activity (Figure 3A) up to 40% at the 4th day of stress above the respective control. Drought gradually raised the activity of SOD up to 230% at the 7th day of stress, but at the 4th day of recovery, it was decreased by 50% above the respective control. The combined treatment (HB + D) caused similar alterations in the leaves of triticale. The activity of SOD progressively increased during the waterlogging treatment up to 40% at the 7th day of stress, but at recovery, it reached the respective control level. The combined treatment (HB + W) caused alterations similar to waterlogging, but at recovery, the SOD activity remained increased by 30% above the respective control.

The herbicide did not cause significant alteration in the CAT activity during the monitored period (Figure 3B). Drought and combined (HB + D) treatment increased the CAT activity by 208% and by 207%, respectively, at the 7th day of stress, but at recovery, the CAT activity was close to the respective control. Waterlogging led to slight increment of CAT activity by 20% at the 4th day of stress only in the combined (HB + W) treatment.

Serrate^®^ increased POX activity (Figure 3C) by 30% only at the 4th day of stress. The activity of POX was increased steadily during drought and combined (HB + D) treatment. The activity was amplified by 588% due to drought and by 558% due to H + D at the 7th day of stress. After 4 days of recovery, it tended to decline but remained increased by 416% and by 428%, respectively, as compared to the control. Although, waterlogging provoked weaker induction of POX activity than drought, the activity rose gradually during the whole monitored period and reached 250% above the respective control at recovery due to both waterlogging and the combined (HB + W) treatment.

The herbicide did not cause significant alteration in GR activity (Figure 3D). Drought increased substantially GR activity at the 4th (by 120%) and 7th (by 360%) days of stress and at recovery (by 110%). A similar increment was found due to the combined (HB + D) treatment. The activity of GR increased slightly (by 20%) at recovery due to waterlogging. The combined treatment (HB + W) raised it by 30% at the 4th day of stress and at recovery. 

### 2.4. Activity of Xenobiotic-Detoxifying Enzymes

**Figure 4:**
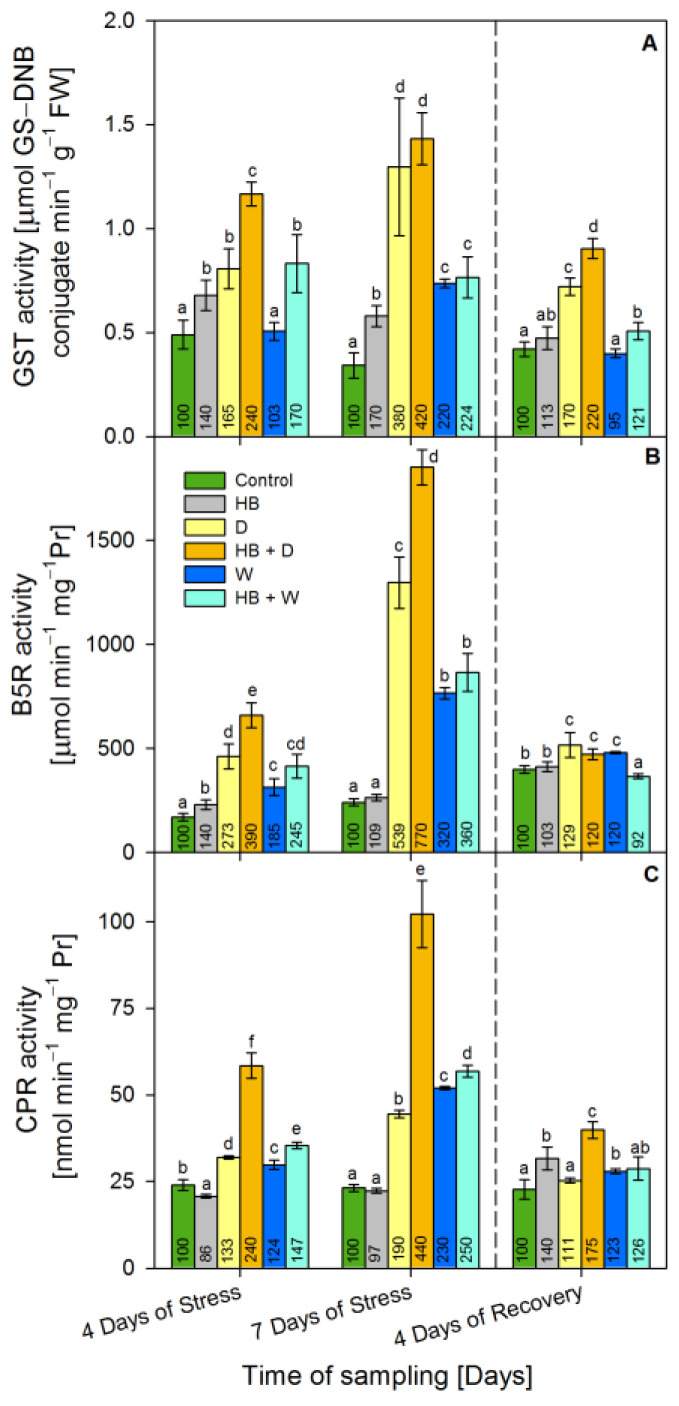
Activity of glutathione *S*-transferase (GST) (GS–DNB: conjugate of GSH and CDNB) (**A**), cytochrome *b5* reductase (B5R) (**B**), and cytochrome P450 reductase (CPR) (**C**) in the leaves of triticale treated with selective herbicide (HB) and exposed to waterlogging (W) or drought (D). The numbers in each bar represent % to the respective controls. The statistical significance between treatments at *p* ≤ 0.05 is indicated by different small letters within the column groups.

The alteration of the GST activity after water stress and herbicide treatment in triticale is presented on Figure 4A. The herbicide raised the activity of GST during treatment (40% and 70% above the control at the 4th day and 7th day of stress, respectively) and reached control values at recovery. Drought increased GST activity by 65% at the 4th day of stress, by 280% at the 7th day of stress and by 70% at recovery above the respective control. The combined treatment (HB + D) raised GST activity even more, by 140% at the 4th day of stress, by 320% at the 7th day of stress and by 120% at recovery above the respective control. Waterlogging provoked a weaker effect on the activity of GST than drought, and it increased the GST activity by 120% only at the 7th day of stress. The combined treatment (HB + W) raised the GST activity by 70% at the 4th day of stress, by 124% at the 7th day of stress and by 21% at recovery above the control.

Serrate^®^ led to a temporary induction (by 40%) of Cytochrome *b5* reductase (B5R) activity only at the 4th day of stress (Figure 4B). Drought increased substantially the activity of B5R by 439% at the 7th day of stress and reverted the activity to 30% over the control at the recovery. The combined treatment (HB + D) further raised B5R activity by 290% at the 4th day of stress, by 670% at the 7th day of stress, but at recovery reverted it to 20% above the control.

Similarly to drought, waterlogging also raised the activity of B5R up to 220% at the 7th day of stress, but at recovery, it was 20% above the control. The combined treatment (HB + W) increased the activity of B5R up to 260% at the 7th day of stress and dropped it slightly (by 8%) below the respective control at the recovery point. 

Serrate^®^ caused slight decline (by 10%) of NADPH:cytochrome P450 reductase (CPR) activity at the 4th day of stress but enhanced it by 40% at recovery (Figure 4C). The activity of CPR gradually increased during drought treatment up to 90% at the 7th day of stress, but after 4 days of recovery it reached the respective control level. The combined treatment (HB + D) raised CPR activity much more—by 140% at the 4th day of stress and by 340% at the 7th day of stress, but the activity decreased to 75% over the control during the recovery stage. Both waterlogging and combined (HB + W) treatment showed similar effect on the CPR activity. The increase of CPR enzymatic activity peaked (by 130% and 150% for waterlogging and the combined treatment) at the 7th day of stress and then dropped close to the respective control at the recovery point. 

## 3. Discussion

Earlier we have published studies concerning treatment combination, applied in a consequent manner–herbicide (Serrate^®^) pretreatment and water stress (drought or waterlogging) in wheat plants [13,14,15,34]. The current study puts triticale on focus because it is expected to be relatively more tolerant to water stress than wheat due to its rye traits, and is an interesting object in the light of the climate changes and the need for more tolerant crops [2,35,36]. Additionally, in real conditions plants are exposed to a number of environmental factors and their effects are not possible to be predicted but just experimentally tested [9,18,19]. Similarly to other environmental challenges, water stress affects ROS and the antioxidative system (enzymatic and non-enzymatic). Under standard growth conditions, ROS and the antioxidative system are in balance and plants keep their cellular redox homeostasis under control [20,21,37]. ROS are implicated in multiple processes as photosynthesis, primary and secondary metabolism, participation in chain reactions, etc., and affect the whole plant’s physiological state. Under abiotic stress conditions, such as water stress, the redox homeostasis is impaired [6]. 

To assess the oxidative stress effects in our model system, the changes in hydrogen peroxide, malondialdehyde and proline content were monitored as well-known plant stress markers. In general, herbicides were reported as a possible cause of oxidative stress and toxicity in various plants [6]. The herbicide Clodinafop, in particular, was reported to increase concentrations of superoxide anion and MDA as markers of lipid peroxidation in leaf discs and in intact 7-day-old winter wheat and rye [38]. The current study also showed a minor induction of stress markers due to the Serrate^®^ application in triticale, which was found at the recovery period. This finding agrees with the consideration that the selectivity of herbicides is not absolute, and they could induce stress responses in tolerant crops as well [12]. The content of the stress markers was observed to be much higher when water stress or the combination treatment (HB + water stress) was applied in triticale. Proline over-accumulation, due to drought and the combined treatment (HB + D) in triticale, was probably linked with cellular toxicity [39,40] because its concentration remains very high even at the recovery. On the opposite, waterlogging, including HB + W, seems to provoke minor increase of stress markers as compared to drought-treated seedlings during the entire experimental period, which confirmed better triticale tolerance to waterlogging than wheat. The herbicide application in combined stress (HB + D and HB + W) did not demonstrate additive negative effects on the oxidative stress markers accumulation in triticale. 

**Figure 5:**
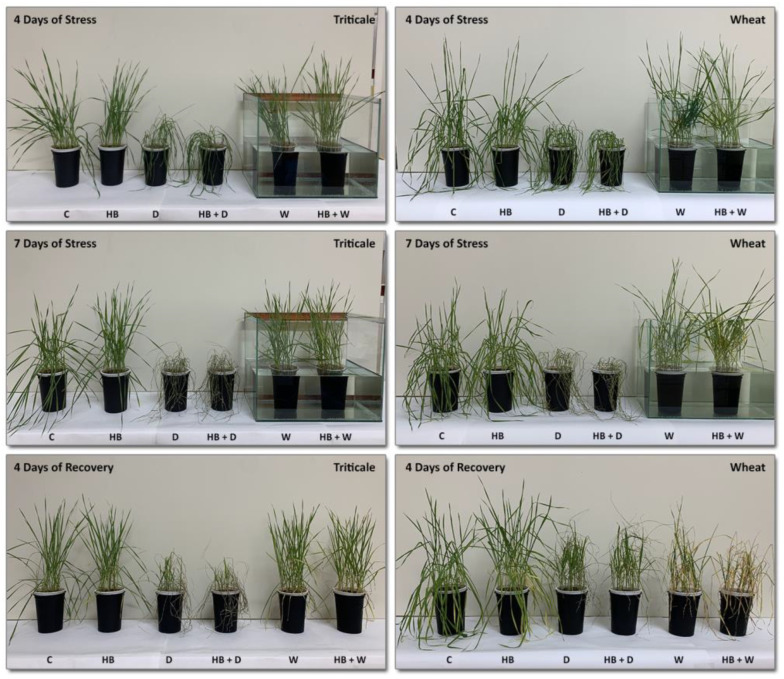
Differences and similarities in phenotype alterations of triticale (left panel) and wheat (right panel—adapted from Todorova et al., 2022 [34]) evoked by herbicide and water stress. C—control; HB—herbicide; D—drought; HB + D—herbicide + drought; W—waterlogging; HB + W—herbicide + waterlogging.

Drought boosted the oxidative stress markers more dramatically than waterlogging and kept that tendency at recovery, pointing to the higher susceptibility of triticale to a water deficit than to waterlogging. Earlier, we reported an opposite effect for wheat plants—waterlogged seedlings were unable to recover at the monitored recovery period, whereas those subjected to drought could recover [13,14,34]. These observations are also well illustrated by the phenotype alterations of triticale as compared to wheat [34] (Figure 5).

Phenolic compounds in plants are part of the secondary metabolites with multiple functions in plants [21,41]. Stress-induced phenolics are predominantly linked with their ROS-scavenging properties, including cell protection from radical chain reactions [42]. Therefore, the slight induction of the total phenolic compounds level due to herbicide application could correspond to the slight induction of lipid peroxidation and hydrogen peroxide. Phenolic compounds in triticale were less altered due to waterlogging as compared to drought treatment. Drought was reported to cause different effects on phenolics content: it does not induce phenylpropanoid biosynthesis in stress-tolerant agave [43]; reduces phenolic compounds in grapevine seedlings [44]; or increases them in amaranth [45]. Hura et al. [46] reported that drought-sensitive triticale cultivars had increased total phenolic content due to water deficit, whereas drought-tolerant cultivars had not. The aforementioned statement is in line with our results showing the highest phenolics’ raise being due to drought and HB + D, and corresponding well with the assumption that triticale cv. Rozhen is more sensitive to drought than to waterlogging. 

One of the most important low-molecular thiol containing compounds is glutathione, which has a major role in stress tolerance, ROS-scavenging, xenobiotic detoxification, regulation of cellular redox status, redox signaling and cross-talk, etc. [21,47,48,49,50]. The slight increase of the low-molecular weight thiols in herbicide-treated plants and in those subjected to waterlogging or to combined treatment (HB + W) might signify the antioxidant and detoxification roles of glutathione. On the contrary, its significant boost due to drought and combined treatment (HB + D) could imply an imbalance of the cellular redox status. The subsequent sharp decrease in low-molecular weight thiols levels at the recovery below the control could also confirm the impaired redox status in triticale plantlets subjected to drought and the combined treatment (HB + D). Such circumstances may hamper triticale plants repairing from drought and the combined treatment (HB + D). The GR enzyme catalyzes the reduction of oxidized (GSSG) to reduced (GSH) glutathione, maintains high the cellular GSH/GSSG ratio and is one of the enzymes of the ascorbate–glutathione cycle, which supports optimal levels of the cellular redox state [21]. Arabidopsis plants overexpressing GR were reported to overcome oxidative stress caused by aluminum via the repression of ROS and the detoxification of lipid peroxide-derived reactive carbonyl species [50]. A fine coordination between non-enzymatic and enzymatic systems is necessary to maintain an optimal redox status in plants [21,47]. For example, although the slight induction of SOD activity due to herbicide application was found, it seems that triticale keeps its redox homeostasis. The increment in H_2_O_2_ due to the Serrate^®^ application at recovery might be detoxified by other peroxidases (such as ascorbate peroxidase) because of the lack of enhanced enzymatic activities of CAT and POX. The moderate induction of SOD, CAT, POX and GR activities, together with the relevant raise of H_2_O_2_ levels imply for good adaptability of triticale after waterlogging, including HB + W. On the contrary, the huge raise of these activities due to drought and HB + D treatment along with the increased stress marker levels during the stress period signify severe oxidative stress events. These observations support our findings that the triticale cultivar Rhozen tolerates waterlogging better than the drought.

Beside typical antioxidant enzymes, we also studied the activity of selected xenobiotic-detoxifying enzymes. The presence of an antidote in the herbicide formulation aims to induce specific metabolic enzymes, which assist the herbicide detoxification, and this could probably be a reason for the enhanced activities of GST, CPR and B5R after the herbicide application. The same explanation could be valid at least partially for the increased activities of detoxification enzymes (GST, CPR and B5R) due to the combined treatments (HB + D and HB + W) as compared with the activities obtained after water stress only. In addition, the participation of GST, B5R and CPR activities in the antioxidative defense could not be excluded [23,24,50]. Interestingly, the enzymatic activity of both B5R and CPR obtained after HB + D was much higher than that measured after drought. In general, CPR provides electrons from NAD(P)H to cytochrome P450 monooxigenases and participates in a vast number of reactions related to secondary metabolites biosynthesis, including phenolics [51]. Our results suggest that in triticale subjected to HB + D, other secondary metabolites might also be involved, apart from phenolics, because the CPR activity is much higher than after drought alone, though the phenolic levels are similar. Plants usually have many CPR genes and their enzymes are classified in class I (constitutively expressed) and class II (stress-inducible) [52] but the monocotyledonous crops are considered as more resistant to herbicides, which could be mediated by P450 monooxigenases [53]. Therefore, the substantially higher CPR activity in triticale after HB + D compared with droughted plants might be due to the expression of a higher number of CPR isoforms. Conventionally, the electron transport chains (ETC) in the endoplasmic reticulum are considered to be NADH:B5R with cytochrome *b5* as an electron donor and NADPH:CPR with P450 [54,55]. In addition, CPR activity is rather related with secondary metabolites synthesis [56] whether B5R is rather linked with the alteration of biomembrane fluidity by increasing unsaturated fatty acids [27,28]. Some stresses, such as desiccation, have been reported to affect membrane integrity and membrane lipid composition. It is well known that the fluidity of the lipid membrane is determined by the content of unsaturated fatty acids, and the change in their amount can affect the plant’s tolerance to stress. Several studies have shown a relationship between the ability of plants to maintain or increase their unsaturated fatty acid content and their resistance to drought [57,58,59]. It was suggested that various ETC could be formed such as NADH:B5R:cytochrome *b5* or NADPH:CPR:cytochrome *b5* [51,60,61]. Based on their results, Zhao et al. [51] and Wayne et al. [62] suggested that both reductases (CPR and B5R) could operate overlapped functions. Therefore, in the current model system the functions of CPR and B5R might also be interrelated. The smaller alteration in GST, CPR and B5R activities, due to waterlogging and HB + W, in comparison to these after drought and HB + D, imply smaller changes in the secondary metabolites and biomembrane fluidity. 

In conclusion, the selective herbicide Serrate^®^ alone does not harshly alter the physiological status of triticale, evidenced by the changes in antioxidant state (stress markers, oxidative and xenobiotic-detoxification status). As expected, drought and waterlogging boosted the levels of stress markers and non-enzymatic compounds, as well as the activities of the antioxidative and xenobiotic-detoxifying enzymes in triticale. In general, the combined treatments (HB + D and HB + W) caused similar trends in the biochemical parameters as compared to those of the water stresses themselves. The effects were more intense after drought than after waterlogging which could mean that triticale cv. Rozhen is more susceptible to drought than to waterlogging. However, the gene expression analyses of key antioxidant and xenobiotic-detoxifying enzymes will expand our observations.

## 4. Materials and Methods

### 4.1. Model System 

Triticale (×*Triticosecale* Wittm.) cv. Rozhen is a certified Bulgarian triticale cultivar originating from the south region of Bulgaria. The seeds were obtained from the Institute of Plant Genetic Resources “K. Malkov” (Sadovo, Bulgaria). Seedlings were grown in a growth chamber under controlled conditions: 60% relative humidity, at 22/19 °C and with a 16/8 h photoperiod (day/night). Seventeen-day-old plantlets grown as a soil culture were leaf sprayed with the herbicide Serrate^®^ according to the manufacturer’s instructions. After 72 h, part of the triticale seedlings were exposed to drought or waterlogging as described earlier [13,14,15,34]. Drought was achieved by withholding watering for 7 days, while waterlogging was realized by relocating the pots into an outer container filled with water at a level 2 cm higher than the soil. The stress program lasted for 7 days, then the plants were transferred to a normal irrigation regime (recovery period). The leaf plant material was randomized within each treatment group, collected after 4 days and 7 days of water stress and after 4 days of recovery. The sample material was weighed, fixed in liquid nitrogen and kept at −70 °C until analysis.

### 4.2. Biochemical Extraction and Analyses

Leaf material (around 250 mg) was ground in 0.1% cold trichloroacetic acid (TCA) and then centrifuged for 30 min at 15,000× *g*, 4 °C. The resultant supernatant was used for the analyses of stress markers (hydrogen peroxide, malondialdehyde (MDA) and free proline) and non-enzymatic antioxidants (phenolic compounds and free thiol-groups-containing compounds). The concentration of hydrogen peroxide was assessed after the dark-incubation (1 h) of 75 µL supernatant with 75 µL 1 M KI [63]. The absorbance was measured at 390 nm, and the concentration of H_2_O_2_ was quantified by a standard curve. The level of lipid peroxidation was estimated by the concentration of MDA in plant tissues [64]. A half milliliter of supernatant was incubated for 45 min at 100° C with 1 mL 0.5% thiobarbituric acid in 20% TCA. The absorbance of the resulting thiobarbituric reaction products was read at 532 nm and 600 nm. The content of MDA was calculated by using the extinction coefficient (155 mM cm^−1^). Free proline was determined according to Bates et al. [65] with some adaptations. Reaction mixture consisted of 0.5 mL supernatant, 0.5 mL 0.1% TCA, 1 mL concentrated CH_3_COOH, and 1 mL ninhydrin reagent (1.25 g ninhydrin, 30 mL concentrated CH_3_COOH, 20 mL 6M H_3_PO_4_) was incubated on a water bath for 1 h at 100 °C, then the reaction was terminated in ice bath and the absorbance was read at 520 nm. 

The total content of the phenolic compounds was assessed using the procedure reported by Swain and Goldstein [66] with a few modifications. Twenty µL supernatant was incubated for 3 min at room temperature with 130 µL distilled water and 50 µL Folin–Ciocalteu reagent. Next, the reaction was supplemented with 50 µL 1M Na_2_CO_3_ and developed in light for 2 h at room temperature. The absorbance was measured at 725 nm, and the results were evaluated by a standard curve prepared with gallic acid (GA). The content of free thiol-groups-containing compounds was assessed by using the Elman’s reagent [67]. The reaction mixture comprised 40 µL supernatant and 150 µL Elman’s reagent. The absorbance was measured at 412 nm after 10 min incubation at room temperature. 

Roughly 200 mg leaf material was homogenized in cold 100 mM potassium phosphate buffer (pH 7.0 with 1 mM EDTA) plus 1% PVP for the assessment of the activities of antioxidant enzymes and GST. The homogenate was centrifuged at 15,000× *g* for 30 min at 4 °C. Catalase (EC 1.11.1.6) activity was evaluated by the monitoring of H_2_O_2_ degradation [68]. The reaction mixture consisted of 50 µL supernatant, 2.930 mL reaction 0.05 M potassium phosphate buffer (pH 7.0) and 20 µL 6% hydrogen peroxide. The enzymatic activity was assessed by following the degradation of H_2_O_2_ for 1 min at 240 nm. Guaiacol peroxidase (EC 1.11.1.7) activity was assessed by using guaiacol as an electron donor. The reaction mixture comprised 20 µL supernatant, 1.1 mL reaction buffer (0.05 M potassium phosphate buffer, pH 7.0), 360 µL 1% guaiacol and 20 µL 15% H_2_O_2_. The absorbance change was monitored at 470 nm [69]. To measure the activity of superoxide dismutase (EC 1.15.1.1) the inhibition of the photochemical reduction of nitroblue tetrazolium was used. The amount of the enzyme required to inflict a 50% inhibition was defined as one unit of SOD [70]. The activity of glutathione reductase was assessed by following the method described by Smith et al. [71]. The reaction mixture contained 100 µL supernatant, 1.180 mL reaction buffer (0.05M potassium phosphate buffer, pH 7.5, 1 mM EDTA), 20 µL 50 mM DTNB, 0.1 mL 7.5 mM oxidized glutathione and 0.1 mL 1.5 mM NADPH. The reaction was assessed at 412 nm for 60 s. The GST activity with 1-chloro-2,4-dinitrobenzene (CDNB, extinction coefficient 9.6 mM cm^−1^ at 340 nm) as a substrate was determined according to Gronwald et al. [72].

Cold 100 mM potassium phosphate buffer (pH 7.5), containing 1 mM EDTA, 0.2 mM PMSF, 1% PVP and 0.3 M sucrose, was used as an extraction solution during the homogenization of the plant material for the assessment of the enzymatic activities of B5R and CPRs. The homogenate was centrifuged at 15,000× *g* for 30 min at 4 °C. Potassium ferricyanide was used as an artificial electron acceptor for the in vitro B5R (EC 1.6.2.2) activity assay [73]. The rate of reduction was evaluated by an extinction coefficient of 1.02 mM cm^−1^ at 420 nm. Cytochrome *c* was used as an artificial electron acceptor for the in vitro CPR (EC 1.6.2.4) activity assay [73]. The rate of reduction was evaluated by an extinction coefficient of 21.1 mM cm^−1^ at 550 nm. The protein content was determined according to Bradford [74]. The herbicide Serrate^®^ was purchased from a local distributor of Syngenta (Basel, Switzerland). All chemical compounds used for the biochemical analyses were obtained from Sigma-Aldrich, (Saint Louis, MO, USA). The measurements of the stress markers, SOD activity and non-enzymatic antioxidants were performed on a Multiskan Spectrum spectrophotometer with a microplate reader (Thermo Electron Corporation, Vantaa, Finland). The enzymatic activities were measured on a Shimadzu UV-1601 spectrophotometer (Shimadzu, Kyoto, Japan). A refrigerated Sigma 2-16K centrifuge (SciQuip, Wem, UK) was used to obtain the respective supernatants.

### 4.3. Statistical Analysis 

The results presented are obtained from three independent biological experiments with three internal replicates. The statistical significance between the treatments were evaluated using one-way ANOVA with a post-hoc Duncan’s multiple range test (*p* ≤ 0.05). The data in the figures represent the average values ± standard error (SE).

## Data Availability

Not applicable.
